# Biosynthesis of Galactan in *Mycobacterium tuberculosis* as a Viable TB Drug Target?

**DOI:** 10.3390/antibiotics9010020

**Published:** 2020-01-06

**Authors:** Zuzana Konyariková, Karin Savková, Stanislav Kozmon, Katarína Mikušová

**Affiliations:** 1 Department of Biochemistry, Faculty of Natural Sciences, Comenius University in Bratislava, Mlynská Dolina, Ilkovičova 6, 84215 Bratislava, Slovakia; konyarikova1@uniba.sk (Z.K.); savkova2@uniba.sk (K.S.); 2 Institute of Chemistry, Slovak Academy of Sciences, Dúbravská Cesta 9, 84538 Bratislava, Slovakia; chemsksa@savba.sk

**Keywords:** UDP-galactose mutase, galactofuranosyl transferase, GlfT1, GlfT2, cell wall, drug design

## Abstract

While target-based drug design has proved successful in several therapeutic areas, this approach has not yet provided compelling outcomes in the field of antibacterial agents. This statement remains especially true for the development of novel therapeutic interventions against tuberculosis, an infectious disease that is among the top ten leading causes of death globally. Mycobacterial galactan is an important component of the protective cell wall core of the tuberculosis pathogen and it could provide a promising target for the design of new drugs. In this review, we summarize the current knowledge on galactan biosynthesis in *Mycobacterium tuberculosis*, including landmark findings that led to the discovery and understanding of three key enzymes in this pathway: UDP-galactose mutase, and galactofuranosyl transferases GlfT1 and GlfT2. Moreover, we recapitulate the efforts aimed at their inhibition. The predicted common transition states of the three enzymes provide the lucrative possibility of multitargeting in pharmaceutical development, a favourable property in the mitigation of drug resistance. We believe that a tight interplay between target-based computational approaches and experimental methods will result in the development of original inhibitors that could serve as the basis of a new generation of drugs against tuberculosis.

## 1. Introduction

Although much progress has been achieved in the global control of tuberculosis (TB) during recent years, the disease remains one of the top causes of death worldwide and the leading cause of death from a single infectious agent [1]. In 2018, an estimated 10 million people contracted TB, and more than 1.4 million people lost their lives to this disease. Treatment of TB is difficult and lengthy. According to current recommendations, patients with drug-susceptible TB require two months of therapy with four antibiotics (ethambutol, isoniazid, rifampicin, and pyrazinamide), followed by at least four months cure with two drugs (isoniazid, rifampicin). The treatment is complicated by the emergence and spread of resistant strains of etiological agent of the disease, bacterium *Mycobacterium tuberculosis*, which accounts for almost 3.4% of the new cases and 18% of the previously treated TB cases [1]. Thus, the need for new, better anti-tuberculosis drugs and more effective drug regimens is pressing. While all of the currently used TB drugs were discovered by phenotypic screening, the value of computational methods and structure-based approaches in this field is becoming evident [2,3,4]. However, finding novel targets suitable for such an approach is challenging. On the basis of the review of current TB drug targets, Kana et al. (2015) listed important issues that have to be considered in target selection [5], which can help prioritize among about 4000 gene products present in *M. tuberculosis* [6]. They suggest several key properties of an “ideal” drug target, such as its essentiality for survival under different physiological states that *M. tuberculosis* encounters during infection, its low inherent mutability, its capacity to serve as a “chokepoint” by affecting multiple processes in the cells, or its essentiality for dormancy. Additionally, an optimal target should be “druggable”, assays should be accessible to assess the catalytic activity and inhibition of the enzyme, and methods should be available to evaluate if the drug remains on-target in whole cells [5].

**Figure 1:**
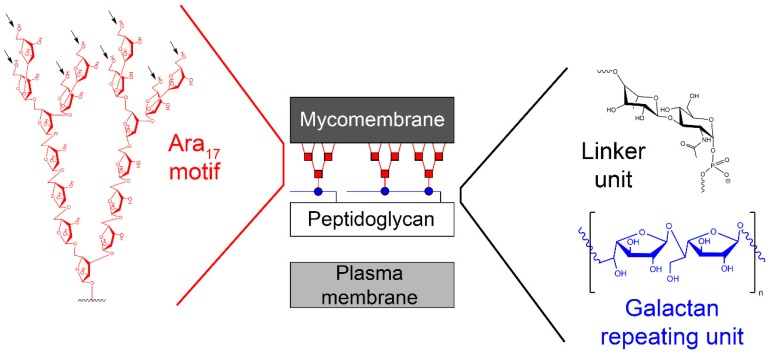
Schematic representation of the cell envelope of *Mycobacterium tuberculosis*. The outermost layer, the capsule, is not shown. Sites for attachment of mycolic acids are indicated by arrows.

One of the validated targets for this new class of anti-tuberculosis compounds, which fulfils the aforementioned criteria, is mycobacterial cell wall construction. Indeed, two medicines of the basic four-drug regimen, isoniazid and ethambutol, affect the biosynthesis of key components of the cell wall core. Moreover, several TB drugs in development target cell wall synthesis in the pathogen [7]. The unique feature of the mycobacterial cell wall is the presence of the mycolyl–arabinogalactan–peptidoglycan (mAGP) complex, which forms an unusual lipidic and extremely hydrophobic barrier that protects the pathogen against the immune system of the host or against common antibiotics [8] (Figure 1). Isoniazid and ethambutol inhibit the production of mycolates [9] and arabinan polymer [10], respectively; however, no current drugs target the synthesis of the galactan core. Owing to the fundamental role of this polymer in keeping the integrity of the cell wall [11], the enzymes that catalyze galactan biosynthesis should be considered as potential candidates for novel drug development. In this review, we present the available information on the galactan component of the mycobacterial cell wall in a historical context; its structural characterization, discovery of the metabolic pathway, and the key enzymes involved in galactan polymerization, as well as a summary of the efforts towards their inhibition. Our aim is to provide inspiration for state-of-the-art target-based approaches [4], which were already successfully applied for the development of potent inhibitors against selected enzymes from *M. tuberculosis* [12,13].

## 2. Structure of the Galactan Component of the Mycobacterial Cell Wall Core: From History to Current Understanding

One of the first reports describing the presence of galactose in the mycobacterial hydrolysates dates back to the 1930s [14]. Later, it was proposed that galactose is a part of a so-called “lipoid-bound polysaccharide” from *M. tuberculosis* [15]. These initial studies were followed by numerous attempts to structurally characterize the basic polysaccharide components in mycobacteria (reviewed in [16]). Among them is the report by Misaki et al. (1966) [17], who fractionated *Mycobacterium bovis* BCG with a series of organic solvents and obtained an insoluble residue containing alanine, glutamate, diaminopimelic acid, glucosamine and muramic acid, as well as neutral sugars glucose, galactose, and arabinose. On the basis of a thorough analysis of this material, they concluded that the main polysaccharide of the mycobacterial cell wall is a highly branched arabinogalactan (AG), which is covalently linked to peptidoglycan (PG) [17]. Identification of the d-arabinose-5-mycolate in various mycobacteria by different research groups led Kanetsuna (1968) to propose that “the mycolic acid–arabinogalactan–mucopeptide complex may be a common structure of mycobacterial cell walls” [18]. The key findings towards understanding the primary structure of this peculiar macromolecular assembly emerged from seminal studies conducted in the late 1980s and in the early 1990s by Brennan, McNeil, and their collaborators. They discovered the nature of the linkage between the AG and PG formed by a disaccharide composed of l-rhamnose (Rha) and *N*-acetyl-d-glucosamine-1-phosphate (GlcNAc-1-*P*), which joins the galactan region with C-6 of *N*-glycolyl/*N*-acetyl muramic acid residues of PG [19]; they confirmed that both arabinose (Ara) and galactose (Gal) are in their furanoid form [20] and defined the basic structural features of the galactan and arabinan motifs, including the mode of the attachment of mycolic acids to the arabinan domain [21,22,23]. Technological development over the last 15 years allowed further clarification of the remarkable features of mAGP, including the localization of minor, but physiologically important substituents, succinate and *N*-acetyl–galactosamine, on the arabinan chains [24,25,26,27]. Today, it is anticipated that the mycobacterial cell wall is a dynamic structure, which is constantly remodelled during the infection process [28,29]. However, changes in the cell envelope organization, under different physiological states, were also reported under laboratory conditions [30]. The latest revised model of the primary structure of AG in *M. tuberculosis* describes the galactofuran polymer composed of an estimated 22 alternating β-(1→5)- and β-(1→6)-linked-d-galactofuranosyl (d-Gal*f*) residues [25], while previously reported values ranged from 30 to 40 galactofuranoses [23,24]. Two arabinofuran chains consisting of highly branched Ara_17_ motifs (with Ara residues linked by α-(1→5), α-(1→3), or β-(1→2) glycosidic bonds, as shown in Figure 1) are attached to C-5 of the β-(1→6)-linked d-Gal*f* residues, close to the reducing end, through a variably long (up to 14 units) linear interior region of the arabinan composed of α-(1→5)-d-arabinofuranosyl (d-Ara*f*) residues [25]. About two-thirds of the available sites (C-5 positions of the last and penultimate Ara*f* residues) are esterified by mycolic acids, always forming clusters of four mycolates per terminal branched pentaarabinoside motif [22] (Figure 1). The current view on the structure of the mycobacterial cell envelope, based on cryomicroscopic studies, suggests the presence of periplasm between the plasma membrane and an outer membrane, or mycomembrane, composed of mycolic acids covalently attached to arabinogalactan and a range of extractable cell wall lipids [31,32].

## 3. Biosynthesis of Mycobacterial Galactan: Discovery of the Metabolic Pathway

**Figure 2:**
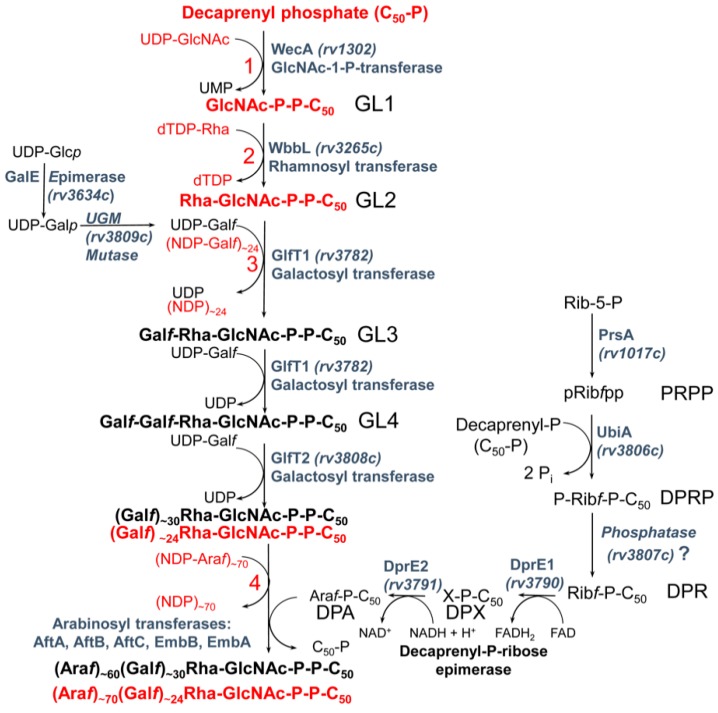
A biosynthetic pathway for the assembly of the arabinogalactan in *M. tuberculosis*. The intermediates and steps 1–4 in red color correspond to those in the original proposal by McNeil and Brennan (1991) [16]. GL1–4—glycolipids 1–4; PRPP—5-phophoribose-1-pyrophosphate; DPRP—decaprenylphosphoryl ribose-5-phosphate; DPR—decaprenylphosphoryl ribose; DPX—decaprenylphosphoryl 2′-keto-β-d-*erythro*-pentofuranose, DPA—decaprenylphosphoryl arabinose.

A hypothetical scheme of mAGP biosynthesis was suggested by McNeil and Brennan in 1991 (Figure 2). The authors claimed the following [16]: “The postulated synthetic routes for biogenesis of cell wall is, of course, highly speculative. None of the enzymes have been purified, and few of the postulated intermediates have been identified in mycobacteria. Nevertheless, elucidation of such pathways is of paramount importance if new effective, tailored, antimycobacterial drugs are to become a reality. The nucleotide sugars involved in arabinogalactan biosynthesis need to be identified, and their biosynthetic pathways need to be elucidated; the presence of the postulated key lipid intermediate Rha-GlcNAc-*P*-*P*-decaprenol must be demonstrated; how Gal*f* residues are added to the putative Rha-GlcNAc-*P*-*P*-decaprenol, whether singularly from a sugar nucleotide donor, or via its own lipid intermediate, must be established. Similar information is lacking about the question of the origins of the Ara*f* residues”.

This proposal was based on the recognition of structural similarities in the linkage unit connecting mycobacterial AG and PG with the junction between PG and cell wall polysaccharides or teichoic acids of Gram-positive bacteria and the key role of these units in the biosynthesis of cell wall structures [33]. It can be stated that today, almost 30 years later, most of the issues raised in this original pathway proposal were resolved (Figure 2). Critical roles in these efforts can be attributed to the publication of the complete genome of *M. tuberculosis* H_37_Rv by Cole et al. already in 1998 [6], as well as the development of techniques that enable genetic manipulation of mycobacterial species, including the isolation of transformable *Mycobacterium smegmatis* mc^2^155 strain [34,35,36].

The first clue suggesting that the hypothetical pathway is likely correct emerged from experiments in which the putative metabolites glycolipid 1 (GL1, polyprenyl-*P*-*P*-GlcNAc) and glycolipid 2 (GL2, polyprenyl-*P*-*P*-GlcNAc-Rha) (Figure 2) were identified as products of the mycobacterial membranes, reacting with the radioactive substrate UDP-[^14^C]GlcNAc [37]. These metabolites were extracted from reaction mixtures with organic solvents and analysed by thin layer chromatography (TLC) followed by autoradiography. Sensitivity of these molecules to mild acid analysis and their stability in mild alkali conditions suggested that they are the polyprenol-*P*-based compounds [38,39]. To verify the presence of Rha in GL2, TDP-[^14^C]Rha was synthesized (because this substrate was not commercially available) and used in reaction with UDP-GlcNAc and mycobacterial membranes. The lipids extracted from the reaction mixture were migrated with [^14^C]GlcNAc-labeled lipids and, indeed, production of [^14^C]Rha-labeled GL2 was confirmed. Synthesis of both glycolipids was severely inhibited by tunicamycin, which is known to interfere with the transfer of GlcNAc-1-*P* from UDP-GlcNAc to polyprenol phosphates giving rise to polyprenol-*P*-*P*-GlcNAc [40]. Addition of UDP-d-Gal*p* and the enzyme fraction containing the mycobacterial cell wall into the reaction mixture resulted in the production of even more polar lipids. It was thus concluded that GL2 serves as a basis for galactan polymerization [37]. This was next confirmed in experiments where UDP-d-[^14^C]Gal*p* was used as a tracer. The radioactive substrate was an effective precursor for the polymerization of galactose on the lipid carrier—GL2 [41]. The true donor substrate for this reaction is, in fact, UDP-d-Gal*f*, which is produced from UDP-d-Gal*p* by the action of UDP-galactopyranose mutase (UGM) [42,43]. This enzyme was later included in the reaction mixture, resulting in a substantial increase in the incorporation of radioactive galactose into the polymeric material [41].

Characterization of the lipid-linked galactan polymer turned into a rather challenging task. For obtaining of this material from the reaction mixture, a protocol including several “washing steps” (0.9% NaCl in 50% methanol, 50% methanol, methanol) and extraction of the reaction products with polar solvents TT3 (CHCl_3_–CH_3_OH–H_2_O; 10:10:3) and E-soak (H_2_O–ethanol–diethyl ether–pyridine–concentrated ammonia, 15:15:5:1:0.017) was developed [41]. The lipid-linked galactan polymers were analysed by SDS-PAGE followed by blotting to nitrocellulose membrane and autoradiography. Similar to GL1 and GL2, the extractable galactan polymers were stable in mild alkalic conditions and cleaved in mildly acidic conditions. Mild acid hydrolysis of the [^14^C]-galactan polymers and subsequent gel filtration showed different sizes of products extracted with solvents TT3 and E-soak. The same material was radiolabeled with UDP-[^14^C]GlcNAc, TDP-[^14^C]Rha, and phospho-[^14^C]ribosyl pyrophosphate (*P*-[^14^C]R*PP*, arabinose precursor), which indicated that galactan polymerization occurred on GL2 and that arabinosyl residues are likely part of the molecule. This was further proved by showing the presence of 5,6-linked galactose in the radiolabeled galactan polymer. These experiments verified that the obtained products are true intermediates of AG biosynthesis [41]. In further work, our group (at Comenius University in collaboration with colleagues from Colorado State University) confirmed that Rv3782 is an initiating galactofuranosyl transferase and Rv3808 is a polymerizing enzyme. We named them GlfT1 and GlfT2, respectively [44].

## 4. UGM, GlfT1, and GlfT2—The Three Key Enzymes with Unexpected Properties

### 4.1. UDP-Galactopyranose Mutase and the Origin of Mycobacterial Galactofuranose

Biosynthesis of galactofuranose was first characterized in *Escherichia coli* K-12 strain in 1996 [42]. Examination of the *rfb* region responsible for production of an *O*-antigen with the repeat unit containing β-d-galactofuranose led to the identification of *orf6* as a gene encoding the putative enzyme responsible for synthesis of this monosaccharide. Furthermore, it was proposed that UDP-Gal*p* could be the substrate for this enzyme [45]. Development of a radiometric assay based on HPLC analysis of the deproteinized reaction mixture treated with phosphodiesterase allowed the monitoring of enzyme activity in cell extracts (as described in Section 5.1). The recombinant protein was purified to homogeneity and the activity test proved that a single enzyme is responsible for catalyzing the conversion of UDP-d-Gal*p* to UDP-d-Gal*f*. Accordingly, the gene encoding this enzyme was designated as *glf* [42]. A similar assay was used in the search for the biosynthetic origin of Gal*f* in mycobacteria [43], both in the forward direction, as well as in the reverse direction. The latter approach was preferred because the equilibrium established between UDP-d-Gal*p* and UDP-d-Gal*f* by the enzyme favors the pyranose moiety by over 90% [42]. The homology search based on *E. coli glf*, which allowed identification of a sequence corresponding to the part of an *M. tuberculosis* gene in the MycDB database, was followed by the characterization of a full *glf* gene found on the specific cosmid from a library prepared from *M. tuberculosis* H_37_Rv DNA. The recombinant protein was produced, and its activity was confirmed by a radiometric HPLC assay [43]. Subsequently, it was found that *rv3809c* gene encoding Glf belongs to a possible AG biosynthetic gene cluster, ranging from *rv3779* to *rv3809c* [46].

**Figure 3:**
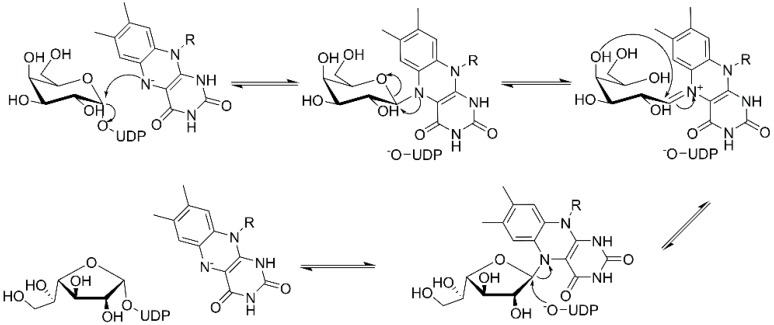
Catalytic mechanism of UDP-galactopyranose mutase (UGM) action. A covalent flavin-galactose intermediate is formed after the nucleophilic attack of the reduced flavin *N*-5 nitrogen to the anomeric *C*-1 galactose carbon employing an S_N_2 process. Interconversion between pyranose and furanose forms occurs via iminium ion.

Examination of the nucleotide sequence of *glf* revealed the presence of a nucleotide binding domain, which was then confirmed experimentally. Indeed, the protein was yellow during purification, and after thermal denaturation, it released FAD [42]. At the time, the role of this cofactor was enigmatic because it was not clear how an oxidation-reduction could be involved in the pyranose-to-furanose interconversion. The structure of *E. coli* UDP-galactopyranose mutase (UGM), solved in 2001 by Sanders et al., revealed the position of the flavin nucleotide in the cleft containing the active site [47]. The isoalloxazine ring of the cofactor was placed close to the sugar of the UDP-Gal*p* substrate, and it was established by the enzyme assays that the protein is active only when flavin is reduced [47]. Numerous substrate and cofactor analogs were examined in order to understand how UGM acts (reviewed in [48]). Finally, the role of FAD in the unique catalytic mechanism of UGM was discovered in 2004 by Kiessling and co-workers [49]. They proposed that N-5 of the reduced flavin serves as a nucleophile, which attacks the anomeric carbon of galactose causing UDP release and formation of a flavin-iminium ion that facilitates sugar ring opening and contraction (Figure 3). To verify this mechanism, they treated radiolabeled UDP-[6-^3^H]Gal*p* with reduced UGM and sodium cyanoborohydride, which would theoretically “trap” the putative intermediate in the enzyme. Subsequent isolation and characterization of a covalent adduct of flavin-galactose confirmed the proposed nucleophilic mechanism [49], recently specified as S*_N_*2-type [50].

**Figure 4:**
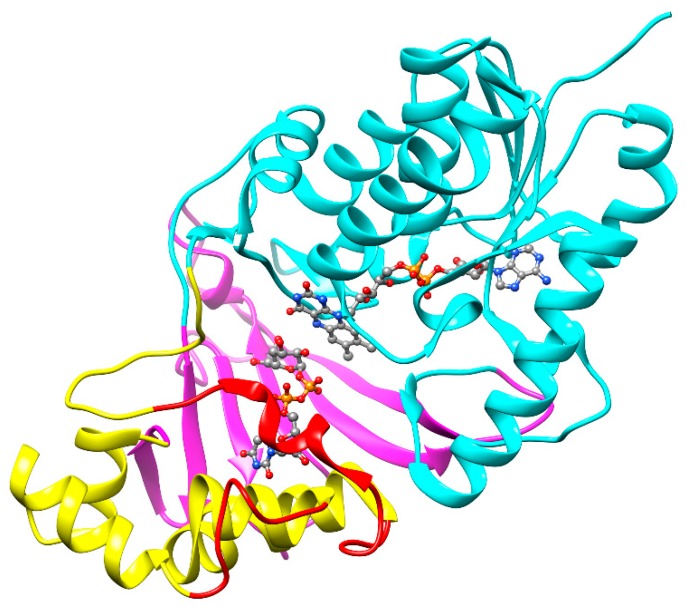
Crystal structure of the UGM*_Mtb_* complexed with UDP-d-Gal*p* and FAD (PDB ID: 4RPG). The monomer unit is composed of the three UGM domains: FAD binding domain-1 (cyan), α-helical domain-2 (yellow), and β-sheet domain-3 (magenta). The flexible loops are represented in red. The UDP-Gal*p* and FAD are shown in the ball-and-stick model.

Numerous structures of UGM both from prokaryotic and eukaryotic organisms have been reported to date [51]. The first structure of UGM from *M. tuberculosis* (UGM*_Mtb_*) in an inactive oxidized state was reported in 2005 by Naismith’s group [52] and, about ten years later, the whole spectrum of the structures, including UGM*_Mtb_* with bound UDP-d-Gal*p* and both oxidized and reduced flavin, as well as the first structure with bound UDP-d-Gal*f* analog (dideoxy tetrafluorinated UDP-F_4_-Gal*f*), was published by Sanders and co-workers [53]. A typical feature of all UGMs, including the *M. tuberculosis* enzyme, is a three-domain architecture (Figure 4). Domain 1 with the Rossmann fold contains the FAD cofactor; the α-helical domain 2 with a mobile loop that reacts to the substrate attachment, binds the uridine group of the substrate; and domain 3, formed from a bundle of six antiparallel β-sheets, contains key residues responsible for interaction with the pyrophosphate. All of the obtained UGM*_Mtb_* structures, liganded or nonliganded and oxidized or reduced, were highly similar to each other and to other prokaryotic UGM structures, with the main difference conferred by ligand binding, which results in active site closure [51,53].

### 4.2. GlfT2—Processive Bifunctional Galactofuranosyltransferase with One Active Site

Early attempts to identify galactosyltransferases involved in AG synthesis through the comparison of the mycobacterial genome with different galactopyranosyl transferase families [54] have failed. We thus turned our attention to *rv3808c* as a candidate gene encoding the potential Gal*f*-transferase, because its product had features typical to other known β-glycosyl transferases in hydrophobic cluster analysis [55], and it overlapped with the *glf* gene (*rv3809c*), encoding UGM, by the first four nucleotides [41]. 

In order to determine the function of the *rv3808c* gene product, we constructed a plasmid for constitutive expression of this gene in *M. smegmatis* mc^2^155. The time course of galactan polymerization in reactions containing enzyme fractions prepared from a recombinant strain producing Rv3808c was compared to that of the control strain. We found that the incorporation of the radiolabel from UDP-[^14^C]Gal*p* into lipid-linked polymers was significantly increased in the case of the recombinant strain. Consequently, we suggested that Rv3808c is a processive galactosyl transferase catalysing the formation of both β-(1→5) and β-(1→6) glycosidic bonds in galactan synthesis [41]. However, the definite confirmation of the dual activity of Rv3808c was presented by Kremer et al. [56]. Synthetic acceptors β-d-Gal*f*-(1→5)-β-d-Gal*f*-*O*-*C*_10:1_ and β-d-Gal*f*-(1→6)-β-d-Gal*f*-*O*-*C*_10:1_ were extended by one or two galactosyl residues by the membrane fractions prepared from an *E. coli* strain that produces recombinant protein Rv3808c. The reaction products were thoroughly characterized by state-of-the-art mass spectrometry, which confirmed the alternating β-(1→5) and β-(1→6) glycosidic bonds [56]. 

The properties of GlfT2 were further investigated after the high-level expression of a soluble recombinant protein in *E. coli* and its successful purification by Lowary and co-workers in 2006 [57]. Activity of the purified enzyme was tested on a range of synthetic acceptors, which confirmed its bifunctional activity and its preference for longer trisaccharide substrates [β-d-Gal*f*-(1→6)-β-d-Gal*f*-(1→5)-β-d-Gal*f*-*O*-*C*_8_ and β-d-Gal*f*-(1→5)-β-d-Gal*f*-(1→6)-β-d-Gal*f*-*O*-*C*_8_] compared with disaccharide ones. MALDI mass spectra of the reaction products with these acceptors revealed the presence of tetrasaccharides, as well as longer products (up to four added residues) and pointed to the faster β-(1→6) Gal*f* transferring activity relative to β-(1→5) activity [57].

Interestingly, when the lipid portion of the β-d-Gal*f*-(1→6)-β-d-Gal*f* containing synthetic acceptors was exchanged to more hydrophobic phenoxy-terminated alkenyl chains of various lengths [–(CH_2_)_1–16_– between the alkenyl and phenoxy moiety], the enzyme was able to catalyze further extension of galactan, up to 48 added galactoses in the case of –(CH_2_)_16_– analog [58]. Surprisingly, even the d-Gal*f* monosaccharide attached to a –(CH_2_)_9_– analog allowed a similar extent of GlfT2 catalysed polymerization, while the d-Gal*p* version of this molecule was completely inactive [58]. The disaccharide series of these acceptor substrates were used to determine the processive character of GlfT2 [59]. The results of these experiments led to a proposed tethering mechanism for galactan length control by Kiessling with co-workers in 2009. The model predicted the existence of a specific site on the enzyme, distal from the active site, which interacts with the lipid portion of the acceptor substrate. The strength of this interaction was proposed to affect the size of the polymer [59]. In follow-up experiments, isotopically labelled acceptor substrate was used to discriminate between the distributive and processive mechanism in a newly developed mass spectrometry assay, which confirmed that GlfT2 is a processive polymerase, that is, staying in contact with the acceptor to perform numerous cycles of catalysis [60].

Recognition of the dual β-(1→5) and β-(1→6) Gal*f*-transferring activities of GlfT2 led to experiments designed to reveal if one or two active sites are engaged in the catalysis. The affinity of the enzyme towards two possible trisaccharide acceptor substrates containing d-Gal*f* linked with alternating β-(1→5) and β-(1→6) glycosidic bonds was examined by saturation transfer difference NMR titration, which pointed to a competition of these substrates for a single active site [61]. This conclusion was supported by identification of the GlfT2 putative active site by homology modelling, in which two conserved DXD motifs characteristic for divergent glycosyl transferases were recognized [62]. The model predicted that GlfT2 belongs to a family of GT-A glycosyltransferases and that both identified motifs of GlfT2, Asp256-Asp257-Asp258, and Asp371-Asp372-Ala-373 play important and distinct roles in catalysis. The former motif was suggested to be involved in the binding of a divalent cation, which is required for the GT-A glycosyltransferases. Mutating the latter motif led to a complete abolishment of both β-(1→5) and β-(1→6) transferring activities, which indicated that it is critical for both catalytic activities of GlfT2 [62]. The crystal structure of GlfT2 resolved in 2012 confirmed that these motifs are, indeed, part of the active site, and that their roles were correctly assigned [63]. 

**Figure 5:**
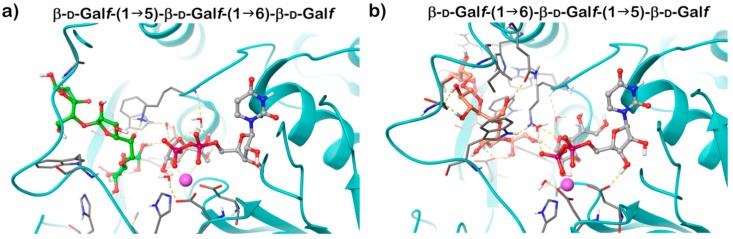
The 3D structures of the modelled Michaelis complexes of the GlfT2 for the β-(1→5) (**a**) and β-(1→6) (**b**) reaction [64]. Contacts between the substrates and the GlfT2 are shown by yellow dashed lines. Mn^2+^ (magenta sphere). Substrates are shown in a ball-and-stick representation.

GlfT2 crystallized in both free and UDP-bound forms and these structures are highly similar, revealing an expected GT-A domain. Modelling of the trisaccharide acceptor substrates with alternating β-(1→5) and β-(1→6) Gal*f* residues and UDP-Gal*f* into the obtained structure explained the dual activity of the enzyme using a single active site. It was proposed that the position of the terminal Gal*f* of the acceptor substrate in the active site affects the production of the next linkage. If the substrate ends with a β-(1→6) Gal*f*, the structure is extended; the last residue is positioned deeper into the active site and directs the new bond in the β-(1→5) position. On the contrary, a shorter length of a β-(1→5) terminated acceptor places the terminal Gal*f* less deep into the active site, which favors the production of a β-(1→6) bond (Figure 5) [63]. The hybrid QM/MM molecular dynamics simulations of the of GlfT2 reaction mechanism suggest that the two reactions proceed in a comparable way and that UDP-d-Gal*f* substrate adopts similar transition states structures [64].

**Figure 6:**
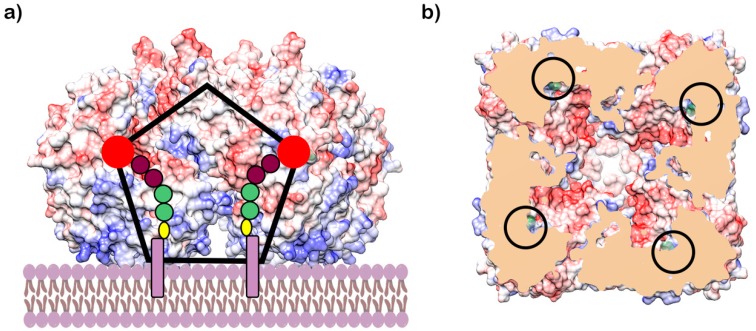
Structure of GlfT2. (**a**) A model of the GlfT2 tetrameric structure sitting on the membrane (PDB ID: 4FIY, bio assembled structure). Approximate schematic position of the UDP-Gal*f* (red circle) and β-d-Gal*f*-(1→5)-β-d-Gal*f*-(1→4)-α-l-Rha*p*-(1→3)-α-d-Glc*p*NAc-decaprenyl-pyro-phosphate during galactan synthesis is shown (Gal*f*—brown, Rha*p* and Glc*p*NAc—light green, pyrophosphate—yellow, and decaprenol—dark pink rectangle). The GlfT2 protein tetramer surface is colored using the electrostatic potential (red—negatively charged, white—neutral, blue—positively charged). The approximate size of the internal cavity is visualized by the black pentagon. (**b**) Sliced view of the GlfT2 tetramer from bottom showing the internal cavity made by quartery structure. Slice is made close to the UDP-d-Gal*p* binding site and the position of the bound UDP-d-Gal*p* is highlighted by black circles.

Interestingly, the protein forms a rather large oligomeric structure, a symmetric homotetramer with a central funnel-shaped pore. The *C*-face of the tetramer contains hydrophobic and positively charged residues, which were proposed to provide the means for the attachment of the enzyme to the membrane (Figure 6) [63]. These unique structural features of GlfT2 led Wheatley et al. to propose that galactan length is controlled by the volume of the central cavity within the tetramer. In fact, it was estimated that the cavity could accommodate about 100–150 Gal*f* residues, which corresponds to the appropriate galactan size of about 25–37 residues per GlfT2 monomer [63]. This model also predicts that most of the lipid portion of the natural acceptor substrate is buried in the membrane rather than interacting with the GlfT2, as proposed for the tethering mechanism for length control [59,63] (Figure 6). Nevertheless, it was suggested that length control is an intrinsic property of a specific GlfT2 enzyme. A recent study by the Kiessling group revealed that the GlfT2 homolog from *Corynebacterium diphtheriae* produces significantly shorter galactan polymers compared with that of its *M. tuberculosis* counterpart in assays containing purified enzymes and alkene-phenoxy acceptor substrates under identical reaction conditions [65]. This finding was explained by the smaller size of galactan in corynebacteria. Nonetheless, on the basis of recent estimates, the galactan size in *M. tuberculosis* [25] approaches that of *Corynebacterium glutamicum* [66].

The first chemical synthesis of natural GlfT2 acceptor substrates containing pyrophosphate and polyprenol moieties was only recently reported by Lowary with co-workers [67]. As revealed previously [44], the natural acceptor-like molecules served as efficient GlfT2 substrates, allowing build-up of a galactan polymer, while their *n*-octyl analogs were extended only by one Gal*f* [67]. The length of the prenyl chain, C_55_ or C_15_, had a minimal effect on GlfT2 activity. This study, in which the activities of purified GlfT1 and GlfT2 were examined with natural-substrate-like acceptors for the first time, provided additional support that GlfT2 serves as a polymerizing galactosyl transferase, while GlfT1 acts as an initiating enzyme [67].

### 4.3. GlfT1—The Enzyme in the Shadow of its More Popular Twin

We initiated studies towards the characterization of the *rv3782* gene product because of the similarity between part of its amino acid sequence and the previously discovered galactosyltransferase Rv3808c, its classification as an inverting nucleotide-sugar requiring glycosyltransferase from the GT-2 family, and its localization in the AG biosynthetic gene cluster of *M. tuberculosis* [68]. In pilot experiments, membrane and cell wall fractions prepared from an *M. smegmatis* strain overproducing Rv3782 were used in a reaction containing UDP-GlcNAc and TDP-Rha for in situ formation of GL2 (decapenyl-*P*-*P*-GlcNAc-Rha) and UDP-d-[^14^C]Gal*p* (as a precursor of UDP-d-[^14^C]Gal*f*). Analysis of the reaction products showed increased synthesis of GL4 (decapenyl-*P*-*P*-GlcNAc-Rha-Gal*f*-Gal*f*) compared with that of the control strain. Similar results were obtained with radioactive GL2 (decaprenyl-*P*-*P*-[^14^C]GlcNAc-Rha) and nonradioactive UDP-d-Gal*f* supplied as the UDP-d-Gal*p* and UGM, or in a reaction mixture containing the enzyme fractions from the control strain supplemented with partially purified Rv3782, in which UDP-d-[^14^C]-Gal*p* was used as a tracer. Hence, the primary product of the reaction was not GL3, which contains only one d-Gal*f* residue, but GL4 with two d-Gal*f* residues. We concluded that Rv3782 is involved in catalyzing the initial steps of galactan synthesis. However, under experimental conditions that employed the crude mycobacterial enzymes, it was not possible to resolve, if Rv3782 catalyzes the conversion of GL2 to GL3, followed by immediate binding of an additional d-Gal*f* via the action of a second endogenous galactosyltransferase present in the reaction mixture, or if the enzyme has a bifunctional activity and catalyzes attachment of both d-Gal*f* residues to the lipid carrier GL2 [68].

Further experiments were designed to accurately determine the functions of Rv3782 and Rv3808c gene products in galactan biosynthesis [44]. At first, we prepared native lipid substrates, GL2-5, radiolabeled with [^14^C]GlcNAc or [^14^C]Gal, respectively, by enzymatic reactions and purified them by preparative TLC. Moreover, we employed synthetic lipid substrates Ac2-5, analogs of natural acceptors containing an octyl chain in place of the decapenylpyrophosphate. Membranes prepared from *E. coli* strains producing Rv3782 homolog from *M. smegmatis* (MSMEG_6367) and Rv3808c from *M. tuberculosis* served as sources of enzymes. In these experiments, we clearly showed that *rv3782* gene product initiates galactan biosynthesis on decapenyl-P-P-GlcNAc-Rha (GL2) acceptor harboring dual β-(1→4) and β-(1→5) d-Gal*f* transferase activity, while *rv3808c* gene product further extends GL4 to produce galactan polymer. Accordingly, we named the two enzymes GlfT1 and GlfT2 [44].

An interesting property of GlfT1 is its donor substrate promiscuity, which became clear when derivatives of UDP-d-Gal*f* modified at C-5 or C-6 (UDP-6-deoxy-6-F-α-d-Gal*f*, UDP-β-l-Ara*f*, UDP-6-deoxy-α-d-Gal*f*, UDP-5-deoxy-α-d-Gal*f*) were examined as potential inhibitors of the enzyme [69]. Cell-free assays with mycobacterial membranes and cell wall fractions revealed that these compounds are, in fact, efficient substrates of GlfT1, which leads to production of short “dead-end” intermediates [69]. A similar property was later described for GlfT2 with UDP-6-deoxy-6-F-α-d-Gal*f* and UDP-5-deoxy-5-F-α-d-Gal*f* substrate analogs [70].

Few publications describe successful, large-scale purifications of GlfT1. In 2008, the production of GlfT1 from *M. tuberculosis* in *E. coli* C41(DE3) was reported using the pET23b expression system. The enzyme transferred a single [^14^C]Gal*f* from the radioactive precursor UDP-d-[^14^C]Gal*p* (in the presence of UGM from *Klebsiella pneumoniae*) on the *n*-octyl β-d-Gal-(1→4)-α-l-Rha acceptor [71]. In the next study published in 2014, a recombinant *M. smegmatis* His_6_-tagged GlfT1 was successfully prepared using an acetamide-inducible mycobacterial expression system based on the pLAM12 vector. The purity of the enzyme was documented by SDS-PAGE analysis [72]. Activity of this enzyme was tested with novel isoprenoid phosphonophosphate disaccharide acceptor substrates that contained l-Rha-α-(1→3)-d-GlcNAc, which mimicks the natural substrate, GL2. The reaction products were identified by MALDI-TOF analysis, which pointed to an extension of the acceptor substrate by two to three Gal*f* residues [72]. 

The availability of the natural acceptor substrate analogs of GL2 and GL3 that contain a farnesyl chain instead of a decaprenyl chain allowed the kinetic characterization of purified GlfT1 enzymes from *M. tuberculosis* and *M. smegmatis*, both produced in *M. smegmatis*, with the use of a spectrophotometric assay (described in Section 5.2) [67]. While each of the enzymes recognized both substrates, the trisaccharide acceptor had an approximately two-fold smaller apparent K_m_ compared with that of the disaccharide acceptor. Remarkably, the products of an overnight incubation of GL2 and GL3 analogs with GlfT1*_Msm_* or GlfT1*_Mtb_* included not only the expected tetrasaccharide, but also more extended oligomers (up to eight Gal*f* residues in the case of the enzyme from *M. tuberculosis* and GL2 analog). However, biological relevance of these observations is not currently apparent. Kiessling with co-workers explained that a tetrasaccharide is the optimal structure efficiently used by GlfT2 [72]. Although galactan disaccharides (or even a substrate with one galactose unit) work as substrates for GlfT2, a kinetic lag phase was observed and attributed to the fact that not all substrate binding subsites are occupied when a shorter acceptor is used. This lag phase was eliminated in lipid-linked Gal*f*-tetrasaccharides that were similar in length to the natural acceptor GL4, produced by GlfT1 [60]. In consideration of these data, GlfT1 appears to play an important role in galactan polymerization control.

## 5. Search for Inhibitors of the Galactan Pathway

**Figure 7:**
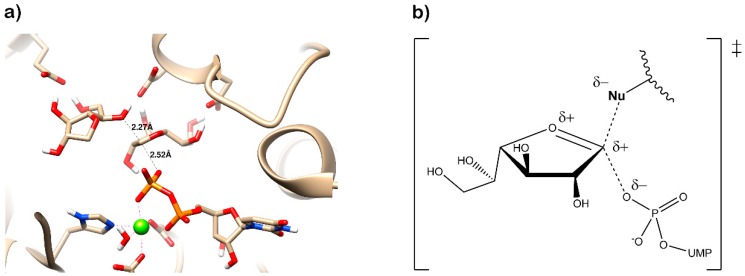
Transition states structures of UGM, GlfT1, and GlfT2. (**a**) A representative structure of the transition state of the β-(1→5) GlfT2 reaction mechanism. The structure was obtained as ensemble averages of the corresponding beads from QM/MM molecular dynamics [64]. Mn^2+^ (green sphere). (**b**) Schematic representation of the probable transition state structure of the GlfT2 reaction mechanism. Represented general scheme can be applied also to the reaction mechanisms of GlfT1 or UGM.

The three core enzymes involved in mycobacterial galactan assembly are all essential for the survival of mycobacteria [11,73] and share the ability to use UDP-d-Gal*f* as a substrate. Because this form of galactose is not present in humans [74,75], these enzymes were repeatedly proposed as potential targets for the development of novel antituberculosis drugs. An attractive option would be to target all three enzymes with one inhibitor, which appears to be relevant owing to a putative common transition state, as recently proposed by Vincent and colleagues [76] (Figure 7).

### 5.1. UGM Assays and Inhibitors

Efforts towards the identification of UGM inhibitors arose soon after its discovery in mycobacteria. The properties of the enzyme, for which the equilibrium favors the UDP-d-Gal*p* substrate over the UDP-d-Gal*f* product by more than 90% [42], as well as the necessity to synthesize commercially unavailable UDP-d-Gal*f* for monitoring the reverse reaction, made the development of the assays applicable for inhibitor screening rather challenging. Although access to sufficient amounts of UDP-d-Gal*f* was limited at the time of UGM discovery, in 2008, Lowary with co-workers published a procedure that yields milligram quantities of UDP-d-Gal*f* [57] by chemoenzymatic synthesis developed by Field and co-workers [77].

The original UGM assay exploited a radioactively labeled UDP-[^14^C]Gal*p* substrate [42]. The reaction mixture was treated with phosphodiesterase I and sugar phosphates released from the substrate or the product, respectively, could then be efficiently separated by HPLC. The assay was also used in the reverse direction, utilizing UDP-[^14^C]Gal*f* substrate [42,43]. In addition, a direct UGM assay that monitors intact sugar nucleotides and can be used with radioactive or non-radioactive substrates was developed [78]. Again, the quantities of the UGM substrate and product were monitored by HPLC, preferentially after the reaction proceeding in the reverse direction, from UDP-d-Gal*f* to UDP-d-Gal*p*. This assay was used to assess the activity of pyrrolidine analogues of galactofuranose on UGM from *E. coli* K12 [79] (Table 1, Entries 1–2).

Further attempts focused on the development of assays suitable for medium to high-throughput screening of compound libraries to identify potential UGM inhibitors. In 2003, McNeil and co-workers exploited the fact that periodate oxidation of UDP-d-[6-^3^H]Gal*f* releases radioactive uncharged formaldehyde after the cleavage of the C-5–C-6 bond, while UDP-d-[6-^3^H]Gal*p* retains the radiolabel after oxidation and remains negatively charged [80]. The radioactive neutral formaldehyde was separated from the charged components of the reaction mixture by the Dowex-1 × 8 anion exchanger and quantified by scintillation counting. The assay was adapted to the 96-well format and used for the screening of a small chemical library of about 1,300 compounds. The most efficient inhibitor was a uridine analog **320KAW73**, with the half maximal inhibitory concentration (IC_50_) of about 6 μM. However, this activity did not translate into inhibition of the growth of *M. tuberculosis* (Table 1, Entry 3) [80]. 

In a follow-up study, nitrofuranylamide inhibitor **1** with IC_50_ = 12 μg/mL, also identified in the previously mentioned screen, was explored based on UGM inhibitory activity and a favourable minimal inhibitory concentration (MIC) of 1.6 μg/mL against *M. tuberculosis* (Table 1, Entry 4). Structure–activity relationships (SAR) were studied on 43 compounds of this series, but even the best UGM inhibitors, **10** and **11** (Table 1, Entries 5–6), had IC_50_ and MIC values comparable to the parent compound **1**. Out of the five compounds selected for in vivo testing based on the MIC, selectivity index, UGM inhibition, CLogP, calculated solubility, and protein binding data, only compound **23** (Table 1, Entry 7) showed activity in a mouse model. However, the authors concluded that, because of a high IC_50_ value, which is in contrast with its low MIC, and a lack of activity on other mycobacterial species, the inhibitory effect on *M. tuberculosis* growth originates from a different primary mode of action [81].

Inspired by the successful use of a fluorescent polarization assay for high-throughput screening of inhibitors of glycosyltransferase MurG, which is involved in the biosynthesis of peptidoglycan precursor Lipid II [82], Soltero-Higgin et al. prepared a fluorescent probe by coupling uridine-5′-diphosphohexanolamine to fluorescein isothiocyanate, which was then used in a newly developed UGM fluorescence polarization assay [83]. This assay measures changes in polarization to monitor the displacement of a probe bound to the active site of an enzyme by a competitive inhibitor. As the assay was originally developed for a *K. pneumoniae* enzyme, IC_50_ and affinity of the probe against UGM*_Mtb_* were first determined to confirm its efficacy for screening of *M. tuberculosis* enzyme inhibitors (Table 1, Entry 8) [84].

A compound library containing 16,000 small, presumably cell permeable, molecules was obtained from Chembridge, and an additional 20,000 compounds were purchased from ChemDiv. These molecules were then screened against UGM*_Mtb_* with 0.3% and 0.15% hit rates, respectively [84]. One of the identified ligands, thiazolidinone derivative **6** from this screen (Table 1, Entry 9) was used as a core scaffold to prepare a directed library of 18 compounds. This library was then evaluated against UGM from *K. pneumoniae* and *M. tuberculosis*. Although a few molecules from the tested set showed better K_d_ values compared with that of the original compound **6**, the authors suggested that reactivity with thiols complicates the performance of these compounds in biological studies [84]. Molecules with such properties are currently classified as pan assay interference compounds (PAINS) [85]. 

In order to overcome this obstacle, Dykhuizen et al. (2008) proposed an alternative, 2-aminothiazole scaffold, which is similar to thiazolidinones, but remains unreactive under physiologic conditions [86]. Within the 62 synthesized compounds from this family screened by fluorescent polarization assays against UGM from *K. pneumoniae* and *M. tuberculosis*, 25 ligands of UGM were identified. Both active (11 molecules) and inactive (3 molecules) compounds were evaluated by an agar disk diffusion assay. Only compounds from the former group were shown to inhibit the growth of *M. smegmatis* mc^2^155, and they did not influence the growth of *E. coli*. Five compounds with different inhibitory activities against UGM*_Mtb_* were chosen to determine MIC in *M. smegmatis*. The value for the most effective inhibitor (Table 1, Entry 12) was 50 μM, but importantly, a correlation between MIC values and UGM inhibitory potency was observed in the selection of five compounds, which suggests that growth inhibition is related to the inhibition of galactan synthesis [86].

The activity of this compound (Table 1, Entries 12, 15) in several mycobacterial species was further evaluated by Borelli et al. (2010), who concluded that it does not inhibit the growth of *M. tuberculosis* up to 100 μg when examined by the disk sensitivity assay [87]. On the contrary, pyrazole compound (Table 1, Entry 14) designed as a potential inhibitor of *M. tuberculosis* growth by computational methods [88,89], which was examined in the same manner in this study, was active against the pathogen and showed dose-dependent inhibition zones in the tested range of 2.5 μg to 20 μg. MICs of this molecule were more than 100 μg/mL for *Bacillus subtilis*, *Micrococcus luteus*, and *Moraxella catarrhalis*, and greater than 200 μg/mL for *E. coli*. Meanwhile, the values for *M. bovis* BCG and two strains of *M. smegmatis* were below 7 μg/mL, indicating a specificity of the molecule against mycobacteria. However, toxicity of both of the tested compounds against mammalian cells (THP1-monocytes) was relatively high. The 50% lethal dose (LD_50_) of the pyrazole compound was 50 μg/mL and even higher for the 2-aminothiazole compound [87].

The intriguing observation that the probe employed for the fluorescent polarization assay binds to UGM more efficiently than UDP (K_d_ values for UGM*_Mtb_* were 0.16 μM and 15 μM, respectively), led Kiessling and co-workers to examine the possibility of the secondary-binding site on the enzyme [90]. They have carefully evaluated the contribution of the fluorescein-UDP linker length and chemical composition to UGM binding, as well as affinities of fragments of the parent compound, containing only the linker-UDP, or fluorescein-linker, and a molecule in which fluorescein was exchanged to naphthyl group. On the basis of these studies, the authors concluded that the secondary binding site relevant for enzyme inhibition is, indeed, present in UGM. Afterwards, they designed a novel compound based on the previously identified 2-aminothiazole scaffold [86] that aimed to exploit the secondary binding site. In fact, this compound proved to be one of the most efficient UGM*_Mtb_* inhibitors at the time (Table 1, Entry 13) [90].

The question of the second inhibitor binding site on UGM was recently raised by Shi et al. (2016), who experimentally and computationally analysed the binding of the pyrazole compound (Table 1, Entry 14) with UGM*_Mtb_*. They identified a putative allosteric site within the enzyme by molecular docking and verified its relevance through mutagenesis of the selected amino acids followed by kinetic analyses [91]. 

The results from a fluorescence polarization assay-based screen of more than 320,000 compounds from the Broad Institute identified only one UGM*_Mtb_* inhibitor with IC_50_ > 250 μM (PubChem AID 504439) [92]. This result motivated Kiessling and co-workers to apply computational methods to virtually screen 4.6 million commercially available compounds against the UGM*_Kp_* structure in the substrate-bound, flavin-reduced conformation. A total of 13 highly ranked molecules with docking scores in the top 0.01% of the library were selected for experimental evaluation by an HPLC assay with the same enzyme. Based on a set of criteria, the triazolothiadiazine compound **6** (Table 1, Entry 16) was chosen as the basis for the selection of the next 11 commercially available molecules. Among these, compound **22** was identified as an efficient competitive inhibitor of UGM*_Kp_*, although its activity against the *M. tuberculosis* enzyme and other UGM homologues was lower (Table 1, Entry 17). Nevertheless, co-crystallization of this molecule with the UGM from *Corynebacterium diphtheriae* successfully resulted in the first structure of the UGM in a complex with a small nonsubstrate-like inhibitor. On the basis of careful examination of compound **22**′s mode of binding to the enzyme, five additional commercially available “second generation” analogs were proposed and their activity against UGM*_Kp_* was tested in both fluorescent polarization and HPLC assays. The best inhibitor **30** (Table 1, Entry 18), which caused complete inhibition of UGM*_Kp_* at a 100 μM concentration, was also active against other UGM homologs, including the enzyme from *M. tuberculosis.* Consequently, the most efficient UGM inhibitors were evaluated for their ability to inhibit bacterial growth. Compound **30** inhibited *M. smegmatis* with MIC 20 μM (9.7 μg/mL), as determined by a microbroth dilution assay. Compound **30** was also the most efficient in the disk diffusion assay against *M. tuberculosis*. Meanwhile, none of the tested compounds killed *B. subtilis* or *E. coli*. The cytotoxicity of this compound with HEK293 cells (LD_50_∼100 μM or 47.5 μg/mL) was explained by an elevated aggregation at higher concentrations [92].

In an effort to further improve the properties of the 2-aminothiazole [86] and triazolothiadiazine [92] series, Kiessling with co-workers focused on the carboxylate part of these molecules and argued that carboxylate replacement with a non-charged functional group surrogate would lead to better mycobacterial penetration. Indeed, 2-aminothiazole compounds modified with *N*-acylsulfonamide showed similar potency at inhibiting UGM*_Mtb_*, but were more efficient inhibitors of mycobacterial growth compared with the original molecule (Table 1, Entry 12 vs. Entries 19–26) [93]. Furthermore, the concentration of 2-aminothiazole derivative **2** (Table 1, Entry 19) in *M. smegmatis,* as determined by LC-MS, was about 14-times higher compared with its carboxylate counterpart (Table 1, Entry 12) [93].

The attractive approach for drug design is to develop transition state analogs. The UGM catalytic mechanism (Figure 3) indicates the possible presence of an oxocarbenium ion in the transition state. Hence, Pinto and co-workers synthesized two mimics carrying a permanent positive charge, 2-deoxy d-arabinitol derivatives containing sulfonium and selenonium ions with an appended polyhydroxylated side chain (Table 1, Entries 29–30) [94]. Similarly, Vincent and co-workers designed analogs of UDP-d-Gal*f* that emulate the transition state of this sugar nucleotide within the active sites of UGM, GlfT1, and GlfT2 (Table 1, Entries 31–32) [76]. However, none of these molecules appeared to efficiently inhibit UGM from *M. tuberculosis*. 

Several recent studies screened libraries of natural products and related compounds to identify further candidate UGM inhibitors. Additional optimization of these molecules through chemical modification [95] or novel compound development through dynamic combinatorial chemistry approaches [96], translated to molecules that cause only moderate inhibition of mycobacterial growth (Table 1, Entries 33–36).

**Table 1:** Inhibitors of mycobacterial UDP-galactopyranose mutase (UGM).

Entry	Year	Compounds Origin	Structure [Reference]	Compound Id ^1^	Assay, Inhibitory Activity	Growth Inhibition
	1997		[79]		HPLC reverse ^2^	
1		structure-based	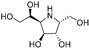	(**1**)	67% at 200 μg/mL	
2		structure-based	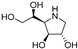	(**2**)	81% at 200 μg/mL	
	2003		[80]		Formaldehyde release ^3^	
3		compound library screening	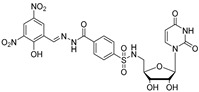	(**320KAW73**)	IC_50_ 6 μM	No activity on *Mtb* in vitro
	2004		[81]		HPLC reverse ^3^	MIC
4		compound library screening	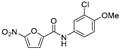	(**1**)	IC_50_ 12 μg/mL	1.6 µg/mL (*Mtb* H_37_Ra)
5		SAR of (**1**)	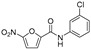	(**10**)	IC_50_ 15 mM	0.8 µg/mL (*Mtb* H_37_Ra)
6		SAR of (**1**)	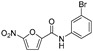	(**11**)	IC_50_ 11 mM	1.6 µg/mL (*Mtb* H_37_Ra)
7		SAR of (**1**)	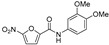	(**23**)	IC_50_ 23 mM	0.2 µg/mL (*Mtb* H_37_Ra)
	2006		[84]		HPLC reverse ^4^	
8		designed probe	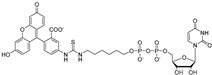	(**1**)	IC_50_ 5.7 μM	
9		compound library screening	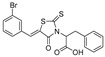	(**6**)	IC_50_ 65 μM	
10		compound library screening	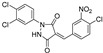	(**9**)	IC_50_ 41 μM	
11		compound library screening	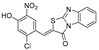	(**10**)	IC_50_ 28 μM	
	2008		[86]		HPLC reverse ^4^	MIC
12		focused library	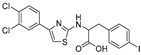		82% at 50 μM	50 µM (*Msmeg*)
	2009		[90]		HPLC reverse ^4^	
13		focused library	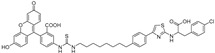	(**10**)	IC_50_ 3.5 µM	
	2010		[87]		Capillary electrophoresis	MIC
14		References [88,89]	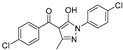	(**1**)	IC_50_ 62 µM	3.3–6.7 µg/mL (*Msmeg*) ^5^ 6.5 µg/mL (*Mbov BCG*)
15		[86]	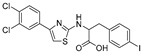	(**2**)	IC_50_ 37 µM	12.5 µg/mL (*Msmeg*) ^5^ 50 µg/mL (*Mbov BCG*)
	2015		[92]		HPLC reverse ^4^	MIC
16		virtual screening	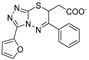	(**6**)	nd	
17		selection based on (**6**)	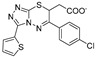	(**22**)	K_i_ 31 ± 18 μM	
18		selection based on (**22**)	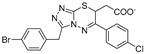	(**30**)	K_i_ 28 ± 15 μM	9.7 µg/mL (*Msmeg*)
	2016	new compounds based on 2-aminothiazole scaffold (Entry 12) [86]	[93] 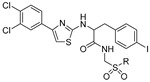		HPLC reverse ^4^	MIC
19			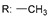	(**2**)	IC_50_ 12 ± 5 μM	25 μM (*Msmeg*)
20			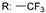	(**3**)	IC_50_ 16 ± 10 μM	25 μM (*Msmeg*)
21			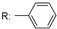	(**4**)	IC_50_ 7 ± 2 μM	12 μM (*Msmeg*)
22			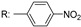	(**5**)	IC_50_ 4 ± 1 μM	50 μM (*Msmeg*)
23			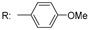	(**6**)	IC_50_ 18 ± 9 μM	25 μM (*Msmeg*)
24			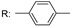	(**7**)	IC_50_ 1 ± 1 μM	12 μM (*Msmeg*)
25			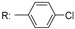	(**8**)	IC_50_ 3 ± 1 μM	6 μM (*Msmeg*)
26			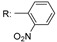	(**9**)	IC_50_ 2 ± 1 μM	12 μM (*Msmeg*)
27		new compounds based on triazolothiadia-zine scaffold	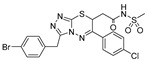	(**12**)	IC_50_ 108 ± 42 μM	
28		(Entry 18) [92]	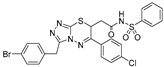	(**13**)	IC_50_ 19 ± 6 μM	
	2016		[94]		HPLC reverse ^4^	
29		mechanism-based	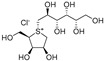	(**1**)	23% at 500 µM	
30		mechanism-based	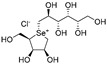	(**2**)	24% at 500 µM	
	2016		[76]		HPLC reverse ^4^	
31		mechanism-based	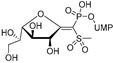	(**46E**)	46% at 500 µM	
32		mechanism-based	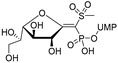	(**46Z**)	23% at 500 µM	
	2017		[95]		HPLC reverse ^4^	MIC
33		natural products screening	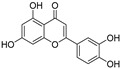	(**4**)	100% at 500 µM	100 µg/mL (*Mtb* mc^2^ 6230)
34		natural products screening	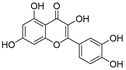	(**3**)	74 ± 8% at 500 µM	>100 µg/mL (*Mtb* mc^2^ 6230)
35		natural products screening	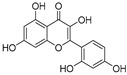	(**28**)	100% at 500 µM	50 µg/mL (*Mtb* mc^2^ 6230)
36	2017	dynamic combinatorial chemistry	[96] 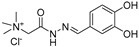	(**H3+A1**)		25 µg/mL (*Mtb* mc^2^ 6230)

^1^ Compound Ids correspond to those used in the original references. ^2^ Source of the enzyme: *E. coli* BL21/pET29 UGM*_Ec_*_K12_ crude enzyme preparation [78]. ^3^ Source of the enzyme: *E. coli* BL21/pET29 UGM*_Mtb_* crude enzyme preparation [43]. ^4^ Source of the enzyme: purified His_6_-UGM*_Mtb_* produced in *E. coli* [84]. ^5^ *M. smegmatis* ATCC 700,084 and *M. smegmatis* ATCC 607 were used.

### 5.2. GlfT1 and GlfT2 Assays and Inhibitors

The first compounds reported to inhibit biosynthesis of mycobacterial galactan were pyrollidine analogues of galactofuranose (Table 1, Entries 1–2). The incorporation of radioactive galactose from UDP-[^14^C]Gal into the polymer catalysed by the crude mycobacterial membrane/cell wall enzyme fraction was determined by descending paper chromatography, in which the polymerized product remained at the start. Compounds **1** and **2** caused 63% and 56% inhibition of galactan polymerization at 200 μg/mL [79]. As the compounds also inhibited UGM from *E. coli*, the authors concluded that they likely act through the inhibition of mycobacterial UGM. A radioactive crude enzyme assay was used to test the next set of iminosugars, which were designed as transition state analogs. None of these compounds caused efficient inhibition of galactan polymerization (Table 2, Entries 1–4) [97,98].

A set of 14 compounds, originally developed as transition state analogs of *E. coli* MurG through docking, were tested against GlfT2 in the assay that exploits the crude mycobacterial cell wall fraction, β-d-Gal*f*-(1→6)-β-d-Gal*f*-*O*-*C*_8_ acceptor and UDP-[^14^C]Gal*p* [99]. In the assay, first described by Kremer et al. in 2001 [56], the neutral reaction products (extended acceptor) are separated from the charged substrate by the strong anion exchange cartridge. Subsequently, the dried eluate is partitioned between *n*-butanol and water. The organic phase that contains the radiolabelled reaction products is then quantified by scintillation counting and further analysed by TLC and autoradiography. The tested molecules were designed as mimics of the oxonium ion formed during the GlfT2 reaction and contained a uridine nucleoside linked through variable spacers to the functionalized proline. One of the tested compounds showed 80% inhibition of the tested enzyme activity at 0.5–1 mM concentration (Table 2, Entry 5) [99].

The successful purification of GlfT2 allowed further modification of the radiometric assay [57]. A trisaccharide substrate β-d-Gal*f*-(1→5)-β-d-Gal*f*-(1→6)-β-d-Gal*f*-*O*-*C*_8_ was identified as an effective acceptor for an assay with the purified GlfT2 and UDP-[6-^3^H]Gal*f* substrate produced in situ from UDP-[6-^3^H]Gal*p* and purified UGM mutase. Separation of the radiolabeled products from the sugar nucleotide substrate was carried out by reverse-phase chromatography on C_18_ cartridges [57].

Access to purified GlfT2 and sufficient amounts of UDP-d-Gal*f* led to the development of the spectrophotometric high-throughput assay with β-d-Gal*f*-(1→5)-β-d-Gal*f*-(1→6)-β-d-Gal*f*-*O*-*C*_8_ used as an acceptor substrate [100]. In this assay, the release of UDP over the course of the GlfT2 reaction is quantified by monitoring NADH consumption linked to coupling reactions catalysed by pyruvate kinase (PK; UDP + phosphoenol pyruvate → UTP + pyruvate) and lactate dehydrogenase (LDH; pyruvate + NADH → lactate + NAD^+^). The assay was adapted for a 384-well format and the kinetic parameters obtained for this trisaccharide acceptor corresponded well to the values measured by the radiometric assay [57,100].

Adaptation of this assay for purified GlfT1 involved the exchange of the trisaccharide acceptor β-d-Gal*f*-(1→5)-β-d-Gal*f*-(1→6)-β-d-Gal*f*-*O*-*C*_8_ for the analog of the natural substrate, GL2, containing farnesol instead of decaprenol [76]. Alternatively, the activity of GlfT1 was monitored spectrophotometrically in the assay with isoprenoid phosphonophosphate disaccharide acceptor substrate containing l-Rha-α-(1→3)-d-GlcNAc, mimicking GL2. In this case, UDP production was linked to a luciferin/luciferase reaction [72].

The efforts towards the identification of GlfT1 and GlfT2 inhibitors focused primarily on the design of transition state or substrate mimics [76,101,102,103,104,105] (summarized in Table 2). The most active among these compounds was fluorinated exo-glycal analogue of UDP-Gal*f* with IC_50_ 180 μM (Table 2, Entry 8), established by a radiometric assay with crude cell wall enzyme fraction from *M. smegmatis* and *O*-alkyl β-d-Gal*f*-(1→6)-β-d-Gal*f* acceptor [102]. Although it is not possible to compare the values obtained by the different assays used in the separate studies, clearly, none of the listed compounds is an efficient inhibitor of the tested enzymes. 

The latest efforts to find GlfT2 inhibitors and to evaluate their properties by molecular docking, 3D-QSAR, and in silico ADMETox studies identified as a potential best candidate thiazolidinone derivative [106], related to the series examined as UGM inhibitors (Table 1, Entry 9), which proved to be PAINS [84,85].

**Table 2:** Compounds designed as substrate mimics or transition state analogs for GlfT1 or GlfT2.

Entry	Year	Target Enzyme	Structure [Reference]	Compound Id ^1^	Assay Inhibitory Activity
	2004	GlfT2	[97]		Radiometric ^2^
1			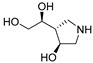	(**10**)	40% at 8 mM
2			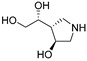	(**11**)	IC_50_ 4.8 mM
	2005	GlfT2	[98]		Radiometric ^2^
3			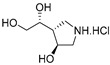	(**25**)	72% at 8 mM
4			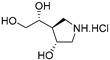	(**3**)	85% at 8 mM
	2010	GlfT2	[99]		Radiometric ^2^ with acceptor
5			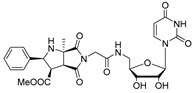	(**16a**)	80% at 1 mM
	2011	GlfT2	[101]		Spectrophotometric ^3^
6			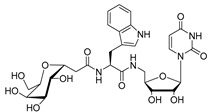	(**6c**)	75% at 4 mM
7			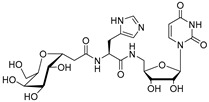	(**6d**)	79% at 4 mM; IC_50_ = 332 μM
	2014	GlfT2	[102]		Radiometric ^2^ with acceptor
					
8			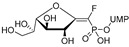	(**E8**)	IC_50_ = 0.18 mM
	2016	GlfT2	[103]		Spectrophotometric ^3^
9			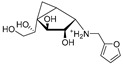	(**4**)	82% at 4 mM
10			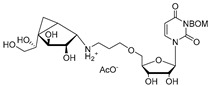	(**29**)	50% at 4 mM
	2016	GlfT1 GlfT2	[76]		Spectrophotometric ^3^
11			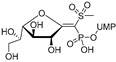	(**46Z**)	33% at 4 mM (GlfT1)IC_50_ 3.85 mM (GlfT2)
12			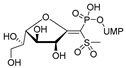	(**46E**)	IC_50_ 1.09 mM (GlfT1)26% at 4 mM (GlfT2)
	2017	GlfT2	[104] 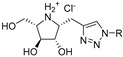		Spectrophotometric ^3^
13			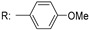	(**9a**)	30% at 4 mM
14			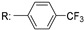	(**9b**)	100% at 4 mM; IC_50_ 0.8mM
15			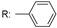	(**9c**)	99% at 4 mM; IC_50_ 0.9 mM
16			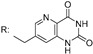	(**9d**)	99% at 4 mM; IC_50_ 1.6 mM
17			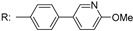	(**9e**)	100% at 4 mM; IC_50_ 2.4 mM
	2018	GlfT2	[105]		Spectrophotometric ^3^
18			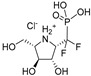	(**3**)	100% at 4 mM; IC_50_ 0.9 mM

^1^ Compound Ids correspond to those used in the original references. ^2^ Source of the enzyme: crude membranes/cell wall from *M. smegmatis*. ^3^ Source of the enzyme: purified GlfT2.

## 6. Conclusions

Galactan biosynthesis has remained an appealing target for novel tuberculosis drug developments since the characterization of this pathway at the turn of this millennium [41,43]. In the early 2000s, conventional belief maintained that an efficient enzyme inhibitor could be developed to the drug by a rational design. Since then, it has become evident, especially in the field of antimicrobials, that this approach is ineffective [107]. In fact, nearly all tuberculosis medications currently in clinical development or recently approved (bedaquiline, delamanid, pretomanid) were discovered by whole-cell screens [7,108]. Despite their commonality, whole-cell screens are limited by the chemical diversity available in the commercial or proprietary libraries and, in the case of UGM*_Mtb_*, this shortcoming can be exemplified by the results of experimental and in silico screening of available compound collections. Nevertheless, we imagine that recent technological advances, both computational and experimental, in the field of tuberculosis drug development [4,12,109,110,111,112,113] provide a promising avenue for therapeutic innovation. Moreover, critical information concerning the galactan biosynthesis pathway has been delineated, including the structures of UGM*_Mtb_* [53] and GlfT2*_Mtb_*[63]. Furthermore, current assays are able to support structure–activity studies. Our summary clearly indicates that only a few candidate compounds were tested in *M. tuberculosis* for growth inhibition (Table 1). Further galactan synthesis inhibitor development should include this information, along with the results of experiments that evaluate whether the drug remains on target in whole cells. These analyses are missing in the previous reports. Varying approaches are currently available for such studies, including the use of overproducers or hypomorphs in the target enzymes. Simple microscopy could also provide the capacity to examine whether the galactan pathway is affected by candidate drugs. Genetic depletion of GlfT2 leads to a specific “lemon-shape” phenotype [114], which could be expected also at target’s chemical inhibition. Alternatively, “old-fashioned” monitoring of cell wall lipid and carbohydrate compositions by metabolic labelling can be applied [10].

Despite the question posed within the title of our review, we believe that, given the attractive possibility of multitargeting of UGM, GlfT1, and GlfT2 by compounds developed as transition state analogs, these enzymes should not be left behind in current efforts towards the development of novel therapeutic interventions against tuberculosis.
